# Vanillin Enhances Photobiomodulation Wound Healing by Modulating Glyco‐Oxidative Stress and Glucose Dysmetabolism in Diabetic Wounded Fibroblast Cells

**DOI:** 10.1111/jcmm.70537

**Published:** 2025-04-07

**Authors:** Ochuko L. Erukainure, Nicolette N. Houreld

**Affiliations:** ^1^ Laser Research Centre Faculty of Health Sciences, University of Johannesburg Doornfontein South Africa

**Keywords:** diabetic wound, glucose metabolism, photobiomodulation, vanillin

## Abstract

Delayed wound healing is among the major peripheral complications of diabetes. Synergistic treatment of diabetic wounds (DW) with phytochemicals and non‐invasive techniques has shown promising results. The synergistic effect of vanillin and photobiomodulation (PBM) on DW healing, and their modulatory effect on oxidative stress and glucose metabolism was investigated in DW fibroblast cells (WS1). DW cells were treated with vanillin and vanillin + PBM. Control consisted of WS1 cells, untreated DW cells, and DW cells treated with PBM. Diabetes was induced by repeated growth in complete MEM containing high D‐glucose (22.6 mM/L). Wounds were induced by central scratching. Cells were treated with vanillin at various concentrations for 2 h prior to PBM at 660 nm with a fluence of 5 J/cm^2^ for an irradiation time of 780 s, followed by 24 h incubation. Induction of DW led to a decreased glutathione level, and decreased superoxide dismutase, catalase, glutathione reductase, glyoxalase, and Na/K‐ATPase activities, while concomitantly increasing the activities of fructose‐1,6‐bisphosphatase, glucose 6‐phosphatase, E‐NTPDase, and 5‐lipoxygenase. These levels and activities were reversed following treatment with 12 μg/mL vanillin, and 6 μg/mL vanillin + PBM having the best effects. However, treatment with 24 μg/mL vanillin and vanillin + PBM showed no significant effects. Except for cells treated with 24 μg/mL vanillin and vanillin + PBM, morphological analysis indicated wound closures compared to the controls. These results indicate the synergistic therapeutic effect of vanillin + PBM on the management of diabetic wounds, with 6 μg/mL vanillin + PBM displaying the best effect.

## 1 Introduction

Diabetic wounds are neuropathic complications of diabetes. They are slow‐healing wounds arising from narrow and reduced blood flow from the body's small blood arteries, particularly those in the extremities (hands and feet). Thus, they cause reduced blood supply of oxygen and nutrients required for healing to the affected tissues, which interrupts the healing process [1, 2]. Poor management of chronic wounds has been associated with impaired mobility, limb amputation, and death [3]. Impaired energy metabolism arising from glucose dysmetabolism due to insulin resistance has been implicated in the aetiology and the pathophysiology of diabetic wounds [4, 5]. Glucose dysmetabolism diminishes the supply of energy for fibroblastic and polymorphonuclear (PMN) activities, thereby impairing wound healing [5]. The glycolytic pathway, which supplies energy to the repairing epithelium, is compromised in diabetic wounds [4, 6]. Instead of the glycolytic pathway, excess glucose is channelled to other pathways such as the polyol, hexosamine, advanced glycation end products (AGEs), and protein kinase C (PKC) pathways, leading to the production of free radicals and inflammatory metabolites which slow the healing process [7]. In a transition‐metal dependent reaction, excess glucose in its enediol form is oxidised to an enediol radical anion, which is converted to reactive ketoaldehydes and superoxide (O_2_˙^−^) anion radicals [8]. Inhibition of the glyoxalase pathway leading to the production of glycosylation end products (AGEs) and accumulation of methylglyoxal from excess glucose has also been implicated in the generation of reactive oxygen species (ROS) from the intermediate metabolite, Amadori compound [9, 10]. Accumulation of methylglyoxal has been linked to the activation of inflammatory cascades which have also been implicated in the pathogenesis of diabetic wounds [11, 12].

There have been major concerns over the management of diabetic wounds as it affects over 15% of people with diabetes [13]. Despite several drugs developed for treating diabetic wounds, there are still concerns about the side effects and costs associated with these drugs. These have led to the continuous search for natural product‐based treatments that are affordable with lesser side effects. Among such natural products is vanillin.

**FIGURE 1:**
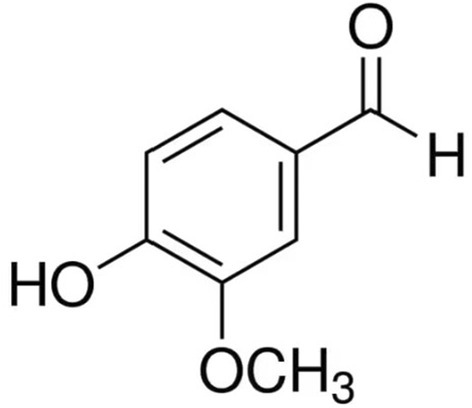
Chemical structure of vanillin.

Vanillin, also known as 4‐hydroxy‐3‐methoxybenzaldehyde (Figure 1), is a phenolic compound and among the most widely used flavours in the food, pharmaceutical, and cosmetic industries with reported antidiabetic, anticancer, antioxidant, and wound healing properties [14, 15, 16]. It is a prominent compound of natural vanilla which is found in mature pods of *Vanilla planifolia* [14]. Vanillin has been reported for lower cytotoxic effects on normal cells compared to other phenolics [17]. The wound healing properties of vanillin have been demonstrated by its ability to stimulate closure of oral mucosal wounds [18]. This is further supported by reports on the protective effect of vanillin against bacteria in skin burns, and its ability to stimulate regeneration and vascularization of the epidermis [16]. Vanillin has been shown to stimulate increased intramuscular vascularization, with concomitant improvement of tibialis anterior and soleus muscle morphology after peripheral nerve injury [19]. Some studies have also reported the effectiveness of chitosan hydrogel crosslinked with vanillin in wound healing [20, 21].

Aside from drugs, photobiomodulation (PBM) has been employed in the treatment of diabetic wounds [22]. It is a non‐invasive technique which utilises photon excitation to modulate biological activities via induction of photochemical reactions which promote wound healing [22, 23]. Its wound‐healing mechanism includes modulation of molecular pathways linked to inflammation, apoptosis, and wound closure [22, 23, 24]. PBM has also been reported for its ability to repair non‐healing ulcers [25], increase collagen synthesis, angiogenesis and granulation [26, 27, 28], and promote reepithelization, cell proliferation, viability, and migration [25].

Studies have shown that combined therapies of PBM and antidiabetic drugs and/or other wound therapies are more effective in the management of diabetic wounds [29]. PBM and metformin showed effective wound healing in type 2 diabetic (T2D) rats [29]. PBM and split‐thickness skin grafting (STSG) have been shown to be an effective cure for burn victims with T2D [30]. A triple combination therapy consisting of PBM, photodynamic therapy, and cellulose membrane has been reported to promote healing in ulcers [31].

Despite these different treatments, there is still a dearth of the synergistic effect of phenolics and PBM on diabetic wound healing. Thus, the present study was aimed at investigating the synergistic effect of vanillin and PBM on diabetic wound healing, and their modulatory effect on key metabolic activities linked to oxidative stress, inflammation, and glucose metabolism in diabetic wounded fibroblast cells (WS1).

## 2 Materials and Methods

### 2.1 Compound

Vanillin (≥ 99.8% HPLC pure) was obtained from Sigma‐Aldrich (Johannesburg, South Africa). A stock solution of 1 mg/mL was prepared using distilled water. The UV absorption of the stock solution was determined using a spectrophotometer.

### 2.2 Cell Line and Groupings

Commercially available human skin fibroblast cells (WS1, ATCC CRL‐1502) were cultured aseptically in accordance with the supplier's instructions.

The cells were grouped into 9 groups, namely NOR = normal cells; DW = diabetic wounded cells (0 J/cm^2^); DWV1 = diabetic wounded cells + 3 μg/mL vanillin; DWV2 = diabetic wounded cells + 6 μg/mL vanillin; DWV3 = diabetic wounded cells + 12 μg/mL vanillin; DWV4 = diabetic wounded cells + 24 μg/mL vanillin; DWVL1 = diabetic wounded cells + PBM (660 nm with 5 J/cm^2^) + 3 μg/mL vanillin; DWVL2 = diabetic wounded cells + PBM (660 nm with 5 J/cm^2^) + 6 μg/mL vanillin; DWVL3 = diabetic wounded cells + PBM (660 nm with 5 J/cm^2^) + 12 μg/mL vanillin; DWVL4 = diabetic wounded cells + PBM (660 nm with 5 J/cm^2^) + 24 μg/mL vanillin; and DWL = diabetic wounded cells + PBM (660 nm with 5 J/cm^2^).

### 2.3 Induction of Diabetes and Wounding

Diabetes was induced in the diabetic groups by continuous growth and several passages in supplemented minimal essential media (MEM) containing an additional 17 mM D‐glucose (total glucose concentration 22.6 mM/L) [32]. The cells were thereafter seeded in 3.4 cm diameter culture plates at a density of 6 × 10^5^ and allowed to attach overnight. A sterile 1 mL pipette was used to create wounds by scratching on a confluent monolayer of cells [33]. The cells were incubated for 30 min at 37°C in a humidified atmosphere with 5% CO_2_ before subjecting them to treatment.

### 2.4 Treatment With Vanillin

The cells were treated with vanillin at different concentrations (3, 6, 12 and 24 μg/mL) as depicted by their grouping and incubated for 2 h prior to PBM.

### 2.5 Laser Irradiation

After incubation for 2 h at 37°C in a humidified atmosphere with 5% CO_2_, the cells were subjected to PBM at a wavelength of 660 nm with a fluence of 5 J/cm^2^ using a diode laser (provided and set up by the Council for Scientific and Industrial Research (CSIR)/National Laser Centre (NLC), Pretoria, South Africa) for 780 s (power output: 60 mW; power density: 6.6 mW/cm^2^; and spot size: 9.1 cm^2^) NOR and DW cells not subjected to irradiation (0 J/cm^2^) were used as controls. Post‐PBM, cells were incubated for 24 h at 37°C in a humidified atmosphere with 5% CO_2_, after which cellular assays were carried out.

### 2.6 Cellular Morphology

The morphology of the cells was assessed with inverted light microscopy (Olympus CKX41 and cell Sens imaging software version 2.3, Wirsam Scientific, Johannesburg, South Africa). Wound closure was estimated by measuring the wound width using ImageJ (https://ij.imjoy.io/). Percentage migration rate was estimated from the wound width using the formula:

$$\%\text{Migration rate}=\frac{\text{width of}\;\mathrm{DW}-\text{width of treated cells}}{\text{width of}\;\mathrm{DW}}\times 100$$

### 2.7 Cell Detachment and Lysis

After 24 h incubation, the cell media was aspirated and the cells were washed with phosphate buffered saline (PBS) before detaching with TrypleExpress. The detached cells were suspended in PBS and lysed by sonicating for 1 min. The lysed cells were centrifuged at 10,000 rpm for 10 min at 4°C. The supernatant was collected and stored at −80°C until further analysis.

### 2.8 Oxidative Stress Parameters

The supernatants were analysed for oxidative stress biomarkers which cover reduced glutathione (GSH) level [34], superoxide dismutase (SOD) [35], catalase (CAT) [36], and glutathione reductase [37].

#### 2.8.1 GSH Level

Briefly, 150 μL of the cells' supernatant was precipitated with an equal volume of 10% trichloroacetic acid (TCA) and centrifuged at 2000 rpm for 10 min at 25°C. Thereafter, 80 μL of the supernatant was mixed with 40 μL of 0.5 mM DTNB in a 96‐well plate. Phosphate buffer (pH 7.8; 200 μL; 0.2 M) was added to the reaction mixture and incubated for 15 min. Absorbance was measured at 415 nm using a microplate reader. The GSH level was extrapolated from a GSH standard graph.

#### 2.8.2 SOD Enzyme Activity

Cell supernatant (15 μL) was mixed with 170 μL of 0.1 mM diethylenetriaminepentaacetic acid (DETAPAC) in a 96‐well plate. Thereafter, 15 μL of 1.6 mM 6‐hydroxydopamine (6‐HD) was added to the mixture. Absorbance was measured at a 492 nm wavelength for 3 min at 1 min intervals.

#### 2.8.3 CAT Enzyme Activity

Cell supernatant (100 μL) was mixed with 1000 μL H_2_O_2_ (65 μM) in 6.0 mM sodium phosphate buffer (pH 7.4) and incubated for 2 min at 37°C. Thereafter, 4 mL of 32.4 mM ammonium molybdate was added to stop the reaction. Absorbance was read at 347 nm against the blank (6.0 mM sodium phosphate buffer (pH 7.4)).

#### 2.8.4 Glutathione Reductase Activity

Briefly, 10 μL of the cells' supernatant was mixed with 221 μL of 50 mM Tris–HCl buffer (containing 1 mM EDTA, pH 8.0) and 38 μL of 8 mM oxidised glutathione (GSSG) in a 96‐well plate. Thereafter, 10 μL of NADPH was added to the reaction mixture. Absorbance was read at 340 nm for 8 min at 2 min intervals using a microplate reader.

### 2.9 Glyoxalase 1 Activity

The glyoxalase 1 (GLO1) activity of the cells was determined using a previously published method [38]. Briefly, 50 μL of 50 mM phosphate buffer (pH 6.6) was mixed with equal volumes of 2 mM methylglyoxal solution and 2 mM reduced glutathione (GSH). The reaction mixture was incubated for 30 min at 37°C. Thereafter, 10 μL of the cells' supernatant was added to the reaction mixture and further incubated for 10 min at 37°C. Absorbance was read 4 times at 240 nm at 2 min intervals using a microplate reader.

### 2.10 Glucose Metabolism

The cells were analysed for glucose metabolism by measuring the activities of glucose 6‐phosphatase and fructose‐1,6‐bisphosphatase in the supernatant [39, 40].

#### 2.10.1 Glucose 6 Phosphatase Activity

Briefly, 200 μL of the cells' supernatant was mixed with 100 μL of 0.25 M glucose 6‐phosphatase, 200 μL of 5 mM KCl, 1300 μL of 0.1 M Tris–HCl buffer, and incubated at 37°C in a shaker for 30 min. Thereafter, 1 mL of distilled water and 1.25% ammonium molybdate was added to stop the reaction. This was followed by the addition of 1 mL of freshly prepared 9% ascorbate. The reaction mixture was allowed to stand for 30 min. Absorbance was then measured at 660 nm.

#### 2.10.2 Fructose‐1,6‐Bisphosphatase Activity

Briefly, 100 μL of the cells' supernatant was mixed with 100 μL of 0.05 M fructose, 1200 μL of 0.1 M Tris–HCl buffer (pH 7.0), 250 μL 0.1 M MgCl_2_, 100 μL 0.1 M KCl, and 250 μL 1 mM EDTA and incubated for 15 min at 37°C. TCA (10%) was added to stop the reaction and centrifuged for 10 min at 3000 rpm (4°C). Thereafter, 50 μL of 1.25% ammonium molybdate and a freshly prepared 9% ascorbic acid were added to 100 μL of the resulting supernatant in a 96‐well plate. The reaction mixture was allowed to stand for 20 min at room temperature. Absorbance was measured at 680 nm.

### 2.11 Purinergic Activities

The cell supernatants were analysed for purinergic enzyme activities which cover Na^+^/K^+^‐ATPase [41] and ectonucleotidase (ENTPDase) [42, 43] activities.

#### 2.11.1 Na^+^/K^+^‐ATPase Activity

Cells supernatant (50 μL) was incubated with 100 μL of Na^+^/K^+^‐ATPase buffer (120 mM Tris–HCl, 0.4 mM EDTA, 200 mM NaCl, 20 mM KCl, and 24 mM MgCl_2_) and 50 μL of ATP for 30 min at 37°C, in the presence or absence of 1 mM ouabain. The reaction was terminated with 50 μL of 10% TCA. Thereafter, 200 μL of 1.25% ammonium molybdate and a freshly prepared 9% ascorbic acid were added to the reaction mixture. The reaction mixture was allowed to stand on ice for 10 min and absorbance was read at 600 nm using a microplate reader.

#### 2.11.2 E‐NTPDase Activity

Briefly, 20 μL of the cells' supernatant was mixed with 200 μL of the reaction buffer (1.5 mM CaCl_2_, 5 mM KCl, 0.1 mM EDTA, 10 mM glucose, 225 mM sucrose and 45 mM Tris–HCl) and incubated at 37°C for 10 min. ATP (50 mM, 20 μL) was added to the reaction mixture and further incubated in a shaker for 20 min at 37°C. 200 μL of 10% TCA was used in terminating the reaction. Thereafter, 200 μL of 1.25% ammonium molybdate and a freshly prepared 9% ascorbic acid were added to the reaction mixture. The reaction mixture was allowed to stand on ice for 10 min and absorbance was read at 600 nm using a microplate reader.

### 2.12 5‐Lipoxygenase Activity

The cell supernatants were analysed for 5‐lipoxygenase activity according to a previously published method [44], with slight modifications. Briefly, 20 μL of the cells' supernatant was incubated with 100 μL of LOX buffer (50 mM Tris–HCl [pH 7.5], 0.3 mM CaCl_2_, 0.1 mM EDTA and 0.1 mM ATP) for 5 min at room temperature. Linolenic acid (40 μL) was then added to the reaction mixture. Absorbance was read at 234 nm 4 times at 30 s intervals.

### 2.13 Statistical Analysis

Samples were analysed thrice (*n* = 3) and statistical analysis was done using SPSS version 27. Data were presented as mean ± SD and considered significant at *p* < 0.05 following Two‐way ANOVA.

## 3 Results

**FIGURE 2:**
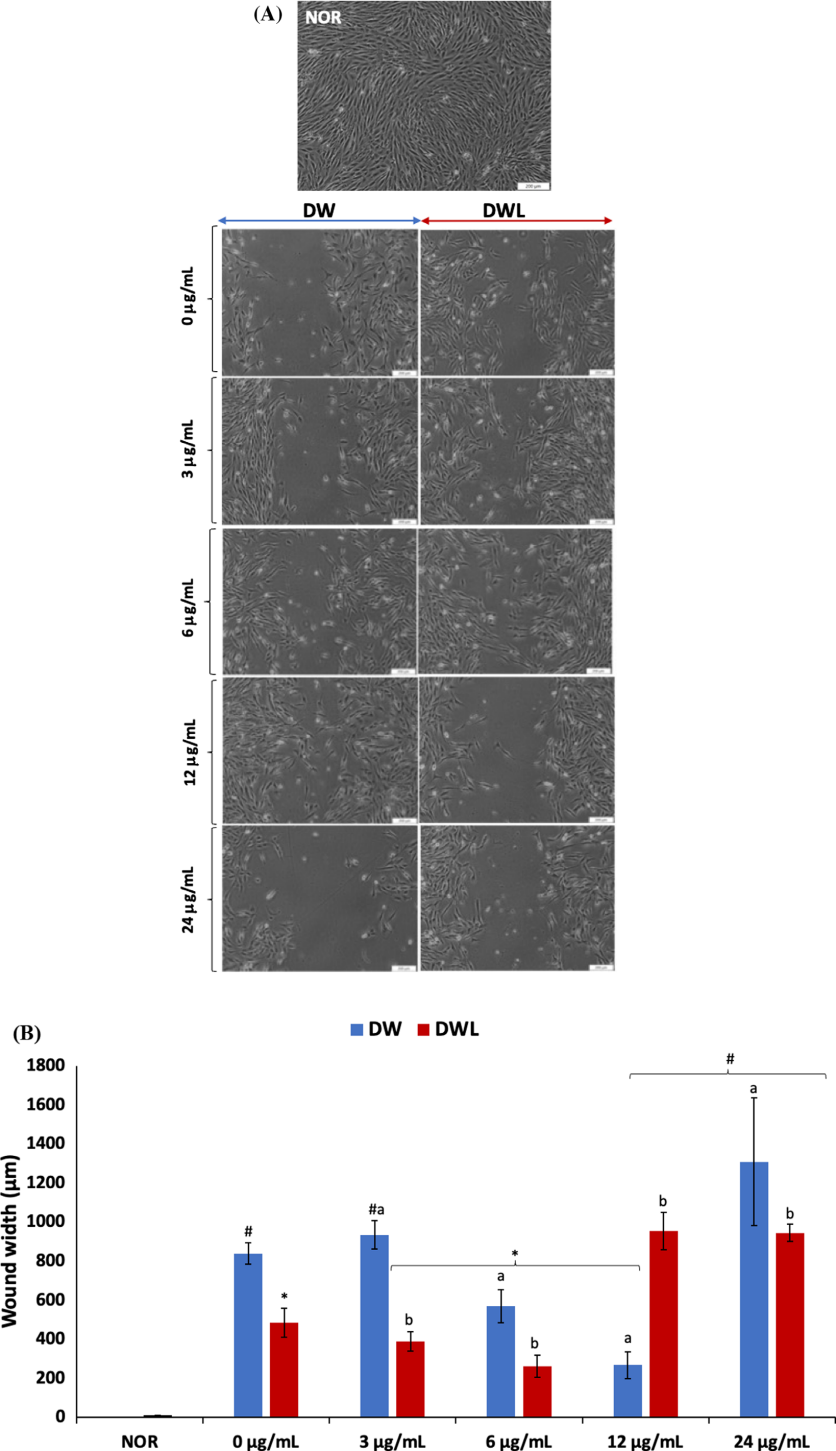
Effect of vanillin and vanillin + PBM (660 nm at 5 J/cm^2^) on (A) cell morphology; and (B) wound width in diabetic wounded fibroblast cells after 24 h treatment. Values = mean ± SD; *n* = 3 for (A, B). Values = mean ± SD; *n* = 3. *Statistically significant (*p* < 0.05) compared to DW; #statistically significant (*p* < 0.05) compared to NOR. ^a,b^Values with different letters above the bars are significantly (*p* < 0.05) different from each other. NOR = normal cells; DW = diabetic wounded cells (0 J/cm^2^); DWL = diabetic wounded cells + PBM (660 nm at 5 J/cm^2^).

As shown in Figure 2, induction of a DW model via a central scratch produced a cell‐free zone indicating a wound, which contrasted with the intact and normal morphology of the NOR cells. Except for DWV4 (24 μg/mL vanillin treatment), treatment with vanillin only led to the increased presence of cells at the borders of the central scratch, with the cell‐free zone partially or completely closed by new cells. The best closure was observed in DWV3 (12 μg/mL vanillin treatment) cells, followed by DWV2 (6 μg/mL vanillin treatment) cells, which are depicted by their reduced wound width (Figure 2B) and % cell migration (68 and 32%, respectively). Cotreatment with vanillin and PBM also led to an increased presence of cells at the borders of the central scratch and closure, which compared favourably to cells treated with PBM only (DWL). The best wound closure was observed in DWVL2 (PBM + 24 μg/mL vanillin treatment) cells (69% cell migration), followed by DWVL1 cells (54% cell migration). For DWV1, DWV4, DVWL3 and DVWL4 treatment groups, the % cell migration was negative, which depicts no wound closure.

**FIGURE 3:**
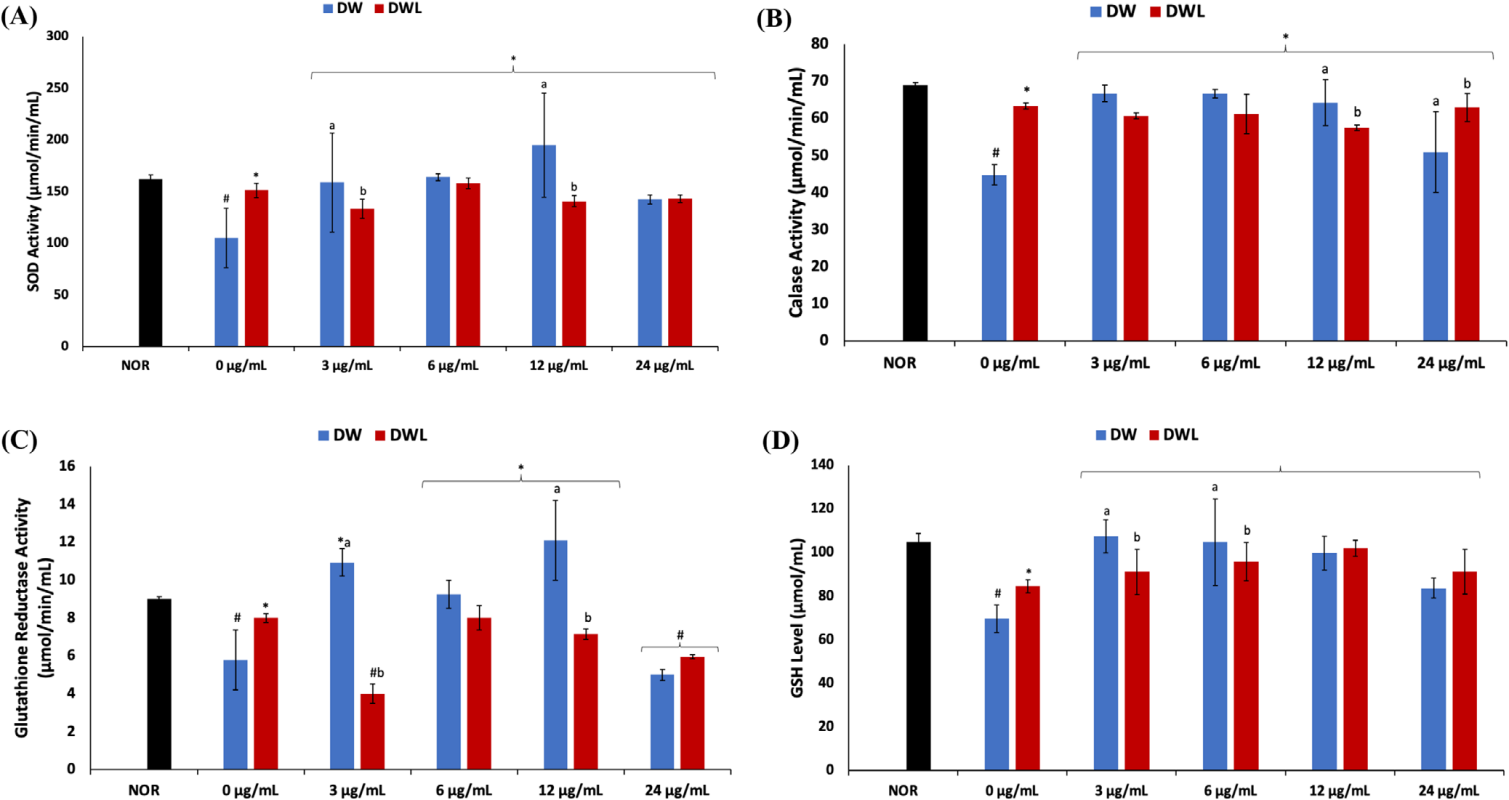
Effect of vanillin alone and vanillin + PBM (660 nm at 5 J/cm^2^) on (A) SOD, (B) CAT, (C) glutathione reductase activities and (D) GSH level in diabetic wounded fibroblast cells after 24 h treatment. Values = mean ± SD; *n* = 3. *Statistically significant (*p* < 0.05) compared to DW; #statistically significant (*p* < 0.05) compared to NOR. ^a,b^Values with different letters above the bars are significantly (*p* < 0.05) different from each other. NOR = normal cells; DW = diabetic wounded cells (0 J/cm^2^); DWL = diabetic wounded cells + PBM (660 nm at 5 J/cm^2^).

As shown in Figure 3A–D, induction of a DW model led to a significant (*p* < 0.05) decrease in SOD, CAT, and glutathione reductase activities, and GSH levels compared to the normal control cells (NOR). These activities and levels were significantly (*p* < 0.05) increased following treatment with only vanillin, except for glutathione reductase activity in DWV4 (24 μg/mL vanillin treatment) cells. These activities and levels were less in WDV4 (24 μg/mL vanillin treatment) cells compared to the other cells treated with only vanillin. Cotreatment with vanillin and PBM also led to significant (*p* < 0.05) increases in SOD, CAT, and glutathione reductase activities, and GSH levels, except for glutathione reductase activity in DWVL1 (PBM + 3 μg/mL vanillin treatment) cells. The increased activities and levels compared favourably with those of cells treated with only PBM (DWL).

**FIGURE 4:**
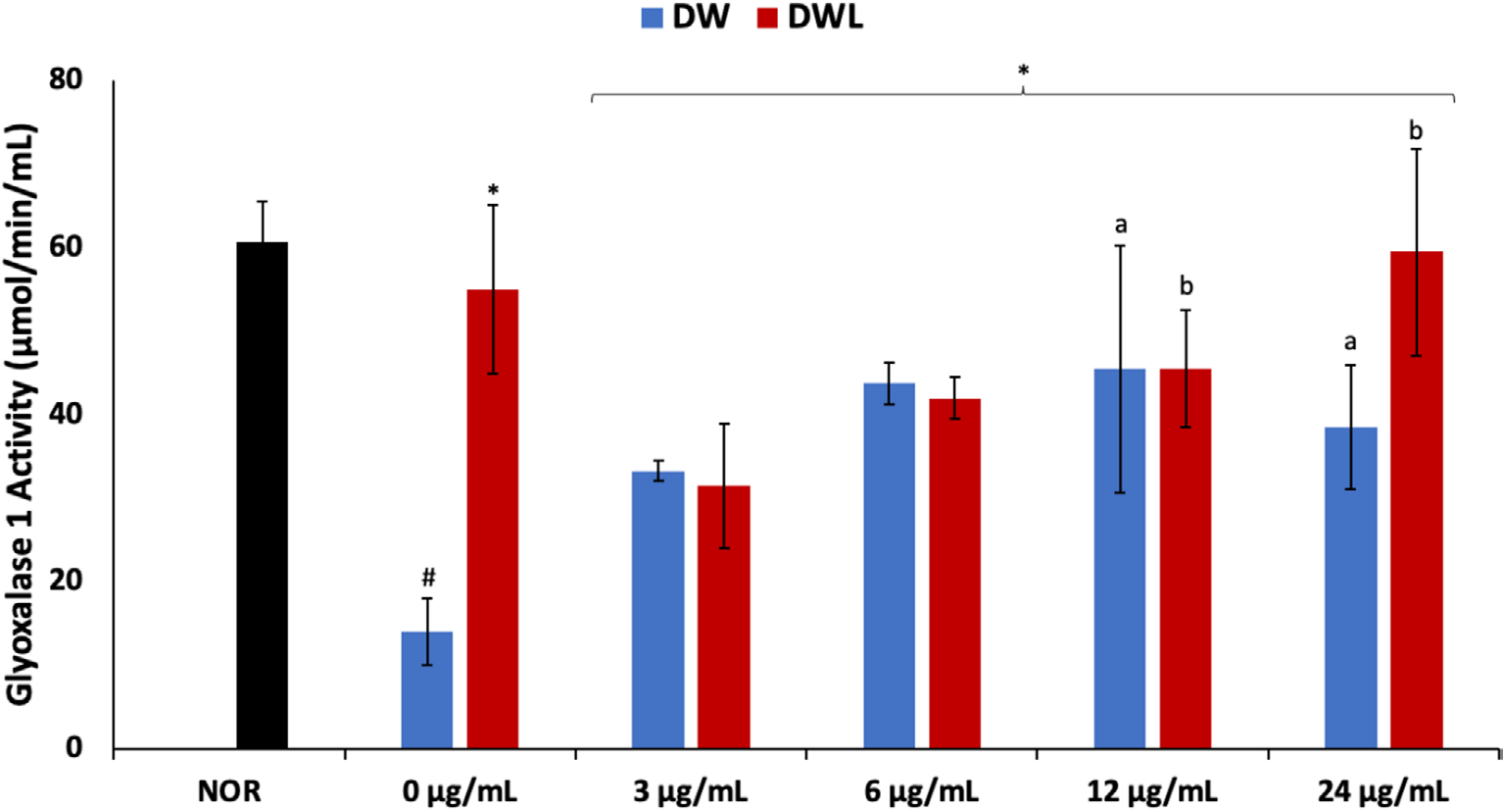
Effect of vanillin alone and vanillin + PBM (660 nm at 5 J/cm^2^) on GLO1 activity in diabetic wounded fibroblast cells after 24 h treatment. Values = mean ± SD; *n* = 3. *Statistically significant (*p* < 0.05) compared to DW; #statistically significant (*p* < 0.05) compared to NOR. ^a,b^Values with different letters above the bars are significantly (*p* < 0.05) different from each other. NOR = normal cells; DW = diabetic wounded cells (0 J/cm^2^); DWL = diabetic wounded cells + PBM (660 nm at 5 J/cm^2^).

There was a significant (*p* < 0.05) decrease in GLO 1 activity following induction of DW compared to the NOR cells, as shown in Figure 4. Treatment with vanillin only significantly (*p* < 0.05) increased the enzyme activity dose‐dependently, peaking at 12 μg/mL. The enzyme activity also increased dose‐dependently in cells cotreated with vanillin and PBM, with DWVL4 (PBM + 24 μg/mL vanillin treatment) cells displaying the highest activity and compared favourably with DWL cells.

**FIGURE 5:**
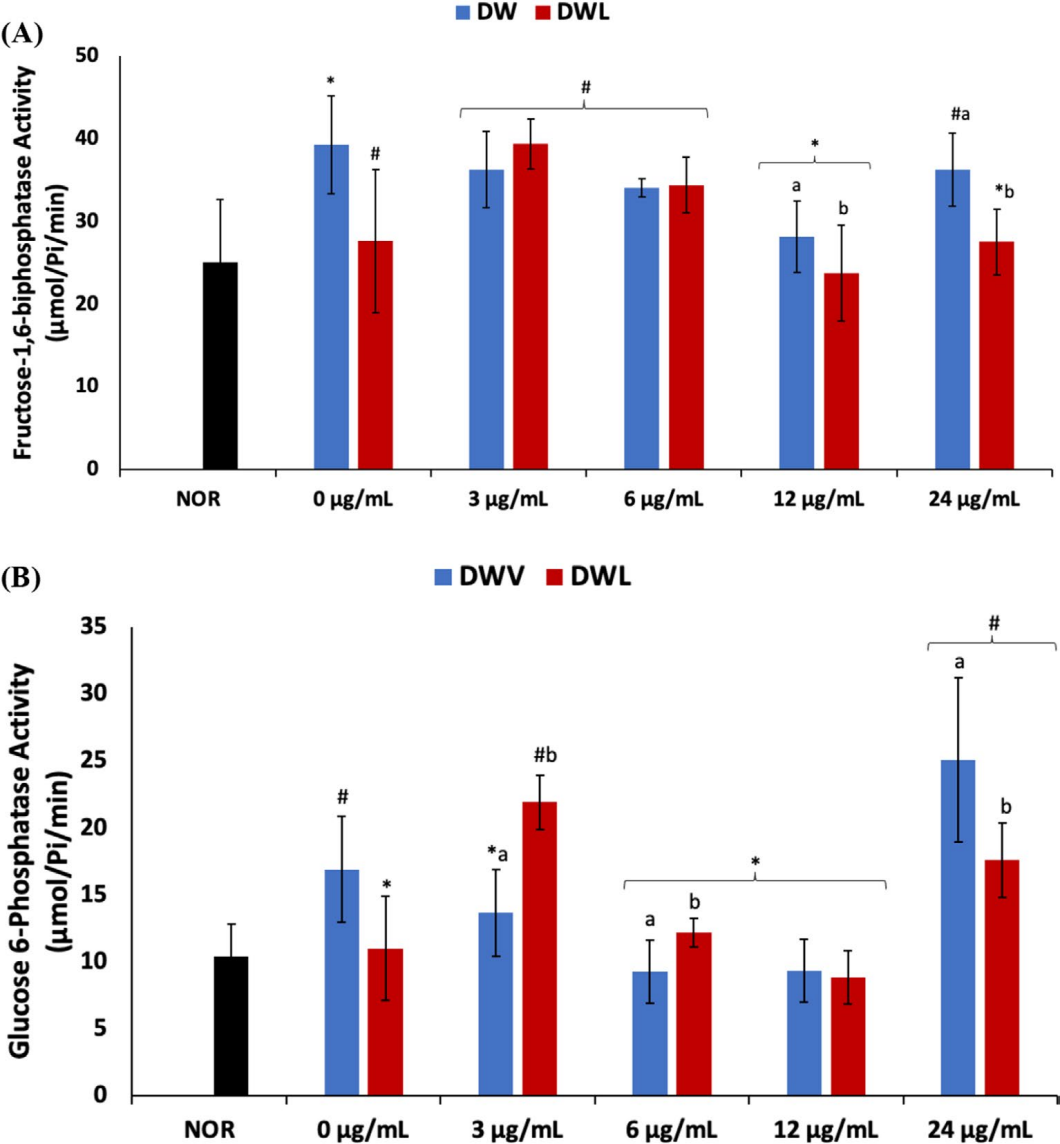
Effect of vanillin alone and vanillin + PBM (660 nm at 5 J/cm^2^) on (A) fructose‐1,6‐bisphosphatase and (B) glucose 6‐phosphatase activities in diabetic wounded fibroblast cells after 24 h treatment. Values = mean ± SD; *n* = 3. *Statistically significant (*p* < 0.05) compared to DW; #statistically significant (*p* < 0.05) compared to NOR. ^a,b^Values with different letters above the bars are significantly (*p* < 0.05) different from each other. NOR = normal cells; DW = diabetic wounded cells (0 J/cm^2^); DWL = diabetic wounded cells + PBM (660 nm at 5 J/cm^2^).

Induction of DW significantly (*p* < 0.05) increased the activities of fructose‐1,6‐biphosphatase and glucose 6‐phosphatase as shown in Figure 5A,B. Treatment with vanillin only led to a dose‐dependent decrease in the activities of these enzymes, except for DWV4 (24 μg/mL vanillin treatment) cells. However, for fructose‐1,6‐biphosphatase activity, the decrease was only significant (*p* < 0.05) in DWV3 (12 μg/mL vanillin treatment) cells. Except for DWVL1 cells, cotreatment with vanillin and PBM led to a significant (*p* < 0.05) decrease in fructose‐1,6‐biphosphatase activity and compared favourably with DWL cells. Cotreatment with vanillin and PBM significantly (*p* < 0.05) decreased glucose 6‐phosphatase activity in DWVL2 and DWVL3 (6 and 12 μg/mL vanillin treatments) cells only.

**FIGURE 6:**
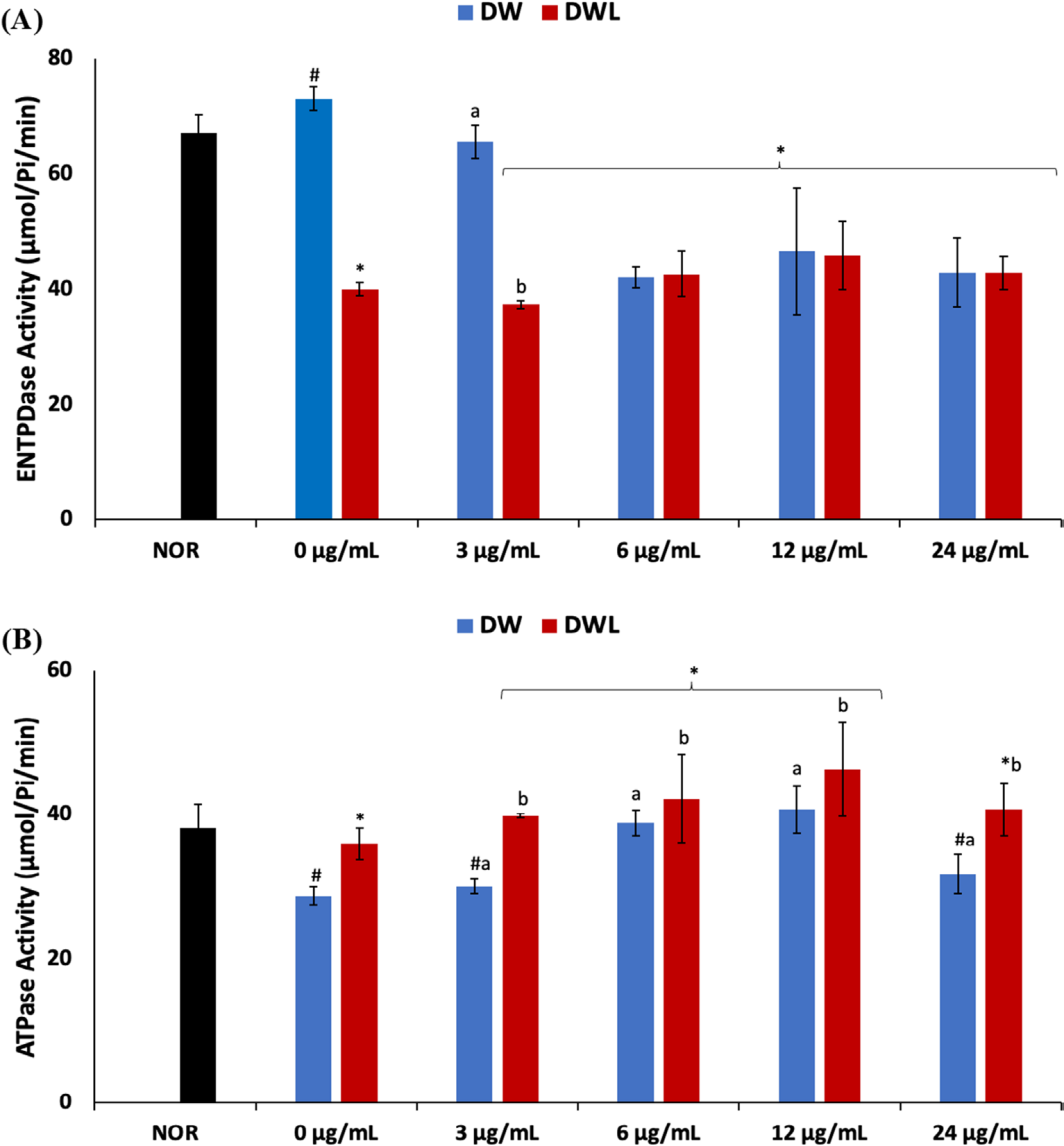
Effect of vanillin alone and vanillin + PBM (660 nm at 5 J/cm^2^) on (A) ENTPDase and (B) Na/K‐ATPase activities in diabetic wounded fibroblast cells after 24 h treatment. Values = mean ± SD; *n* = 3. *Statistically significant (*p* < 0.05) compared to DW; #statistically significant (*p* < 0.05) compared to NOR. ^a,b^Values with different letters above the bars are significantly (*p* < 0.05) different from each other. NOR = normal cells; DW = diabetic wounded cells (0 J/cm^2^); DWL = diabetic wounded cells + PBM (660 nm at 5 J/cm^2^).

There was a slight increase in ENTPDase activity following induction of DW as shown in Figure 6A. Treatment with only vanillin led to a significant (*p* < 0.05) decrease in the enzyme activity in DWV2 (6 μg/mL vanillin treatment), DWV3 (12 μg/mL vanillin treatment) and DWV4 (24 μg/mL vanillin treatment) cells. Cotreatment with vanillin and PBM significantly (*p* < 0.05) decreased the enzyme activity.

As shown in Figure 6B, induction of DW significantly (*p* < 0.05) decreased the activity of Na^+^/K^−^ ATPase. The enzyme activity was significantly (*p* < 0.05) increased in cells treated with only vanillin, except DWV4 (24 μg/mL vanillin treatment) cells, which rather had a decreased enzyme activity. Cotreatment with vanillin and PBM also led to a significant (*p* < 0.05) increase in the enzyme activities, except DWVL4 cells.

**FIGURE 7:**
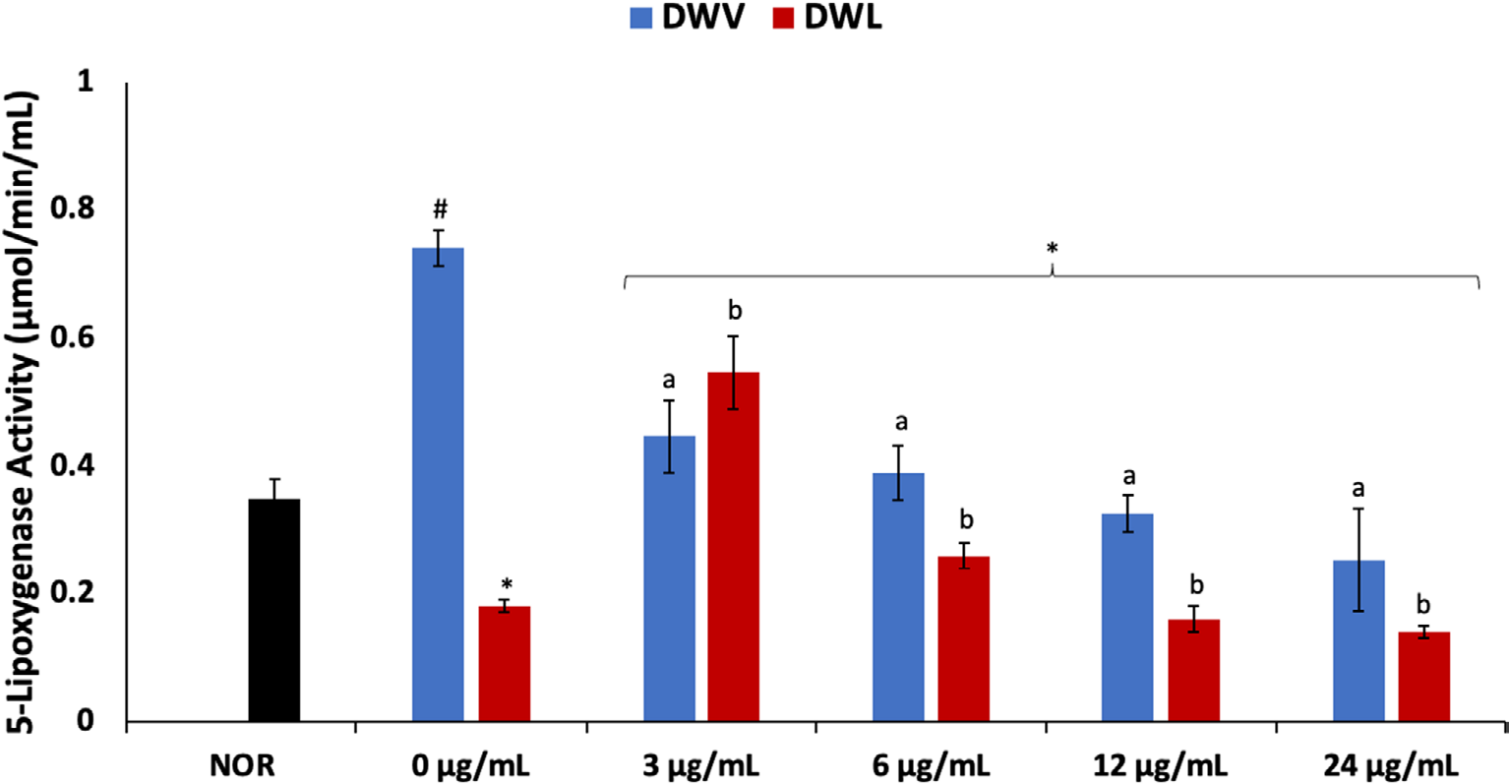
Effect of vanillin alone and vanillin + PBM (660 nm at 5 J/cm^2^) on 5‐LOX activity in diabetic wounded fibroblast cells after 24 h treatment. Values = mean ± SD; *n* = 3. *Statistically significant (*p* < 0.05) compared to DW; #statistically significant (*p* < 0.05) compared to NOR. ^a,b^Values with different letters above the bars are significantly (*p* < 0.05) different from each other. NOR = normal cells; DW = diabetic wounded cells (0 J/cm^2^); DWL = diabetic wounded cells + PBM (660 nm at 5 J/cm^2^).

As shown in Figure 7, there was a significant (*p* < 0.05) increase in 5‐LOX activity following induction of DW as compared to NOR cells. The enzyme activity was significantly decreased dose‐dependently following treatment with vanillin only. Except for DWVL1 (PBM + 24 μg/mL vanillin treatment), cotreatment with vanillin and PBM significantly decreased the enzyme activity dose‐dependently.

## 4 Discussion

Slow healing wounds arising from neuropathic complications of diabetes are major concerns to health practitioners as they contribute to impaired mobility, limb amputation, and motility [13, 45]. There has been an increased search for therapies to enhance wound healing in diabetes, with paradigm shifts toward natural products and PBM. In the present study, the synergistic effect of vanillin and PBM on diabetic wounds was investigated in diabetic wounded WS1 cells. In this study, vanillin improved the wound healing properties of PBM by enhancing wound closure, antioxidant and purinergic activities, and glucose metabolism.

Cellular migration has been linked to wound closure. The cell‐free zone in DW cells following the induction of a wound (Figure 2A) indicates suppressed cellular migration, which is characteristic of diabetic wounds [46, 47]. The presence of cells in the gap (cell‐free zone) in DWV1—3 (3, 6 and 12 μg/mL vanillin treatment), DWVL1–4 (PBM + 3, 6, 12 and 24 μg/mL vanillin treatment), and DWL cells indicates cellular migration (Figure 2), thereby suggesting wound healing and improved migration. This corroborates previous reports on the wound healing properties of vanillin [18, 19, 48]. The high cellular migration in DWV3 (12 μg/mL vanillin treatment) cells may indicate that the optimum concentration at which vanillin may bring about its wound closing effect is 12 μg/mL. Furthermore, the low cellular migration in DWV4 (24 μg/mL vanillin treatment) cells suggests that vanillin may be toxic against diabetic wounds with increasing concentration. The increased cellular migration in DWVL2 (6 μg/mL vanillin treatment) cells (Figure 2) indicates that the optimum concentration at which vanillin may enhance the wound healing properties of PBM is 6 μg/mL.

Oxidative stress has been implicated in diabetic wounds as they contribute to delayed healing owing to elevated cellular levels of ROS, while concomitantly suppressing antioxidant activities [49]. It is characterised by elevated cellular levels of superoxide (O_2_˙^−^) and hydrogen peroxide (H_2_O_2_) and alterations in glutathione metabolism [50, 51]. This is depicted in the present study by the suppressed activities of SOD, CAT, and glutathione reductase, and decreased GSH level in DW cells (Figure 3A–D). SOD catalyses the dismutation of O_2_˙^−^, while CAT hydrolyses H_2_O_2_ to water (H_2_O) and oxygen (O_2_). Thus, the suppressed SOD and CAT activities indicate elevated cellular levels of O_2_˙^−^ and H_2_O_2_. Glutathione reductase catalyses the reduction of oxidised glutathione (GSSG) to GSH, suggesting that their suppressed activity and levels indicate altered glutathione metabolism. The elevated SOD, CAT, glutathione reductase activities, and GSH level in the treated cells indicate suppressed cellular levels of O_2_˙^−^ and H_2_O_2_, and improved glutathione metabolism. Thus, an improved antioxidant activity. This correlates with previous reports on the ability of PBM to modulate oxidative stress biomarkers in diabetic wounds in vivo [52]. Thereby, corroborating previous studies on improved antioxidant activities in diabetic wound healing [53, 54, 55].

Methylglyoxal is a highly reactive dicarbonyl compound produced during glucose metabolism which reacts with proteins, lipids, and DNA to generate AGEs that have been implicated in diabetic vascular complications [56, 57]. Increased accumulation of methylglyoxal with concomitant suppressed GLO1 expression has been reported in diabetic wounds [56]. Glyoxalase 1 is a key metabolic enzyme that catalyses the hydrolysis of methylglyoxal to S‐D‐lactoylglutathione. Thus, the suppressed GLO1 activity in the DW cells (Figure 4) suggests an accumulation of methylglyoxal. The elevated GLO1 activity in the treated cells, therefore, suggests reduced methylglyoxal levels which indicate an improved wound closure [57]. Glyoxalase 1 is also a GSH‐dependent enzyme. Thus, the increased activity in the treated cells corroborates the elevated GSH levels following treatment (Figure 3D). The low activity in DWV4 (24 μg/mL vanillin treatment) cells may suggest a toxic effect of vanillin on diabetic wounds at higher concentrations. The high enzyme activity in DWVL4 (PBM + 24 μg/mL vanillin treatment) cells suggests an improved synergistic effect of vanillin and PBM.

Suppressed glycolysis in diabetic wounds has been implicated in impaired energy supply for fibroblastic and PMN activities required for wound healing [5]. The increased activities of fructose‐1,6‐bisphosphatase and glucose 6‐phosphatase in the DW cells (Figure 5A,B) indicate activation of gluconeogenesis and suppression of glycolysis. Thus, it suggests an increased accumulation of glucose in the cells which compromises the supply of energy for wound healing. The accumulated glucose can be oxidised to O_2_˙^−^ [8] or channelled to other pathways such as the polyol, PKC, and hexosamine pathways to generate methylglyoxal and other inflammatory metabolites which further suppress wound healing [7]. The decreased activities of fructose‐1,6‐bisphosphatase in DWV3 (12 μg/mL vanillin treatment), DWVL3 (PBM + 12 μg/mL vanillin treatment), DWVL4 (PBM + 24 μg/mL vanillin treatment) and DWL cells (Figure 5A) and glucose 6‐phosphatase in DWV1—3 (3, 6 and 12 μg/mL vanillin treatment), DWVL2 (PBM + 6 μg/mL vanillin treatment), DWVL3 (PBM + 12 μg/mL vanillin treatment) and DWL cells (Figure 5B) indicate activation of glycolysis and improved energy metabolism. Thereby, it suggests decreased availability of glucose for the production of O_2_˙^−^ and methylglyoxal. The high activities of these enzymes in DWL4 (24 μg/mL vanillin treatment) may also suggest a toxic effect of vanillin on diabetic wounds at high concentrations. While the low activities in DWVL3 (PBM + 12 μg/mL vanillin treatment)cells suggests an improved synergistic effect of vanillin and PBM.

Increased activity of ENTPDase has been implicated in impaired wound healing in diabetes [58]. The enzyme catalyses the hydrolysis of ATP and/or ADP to AMP, which has been reported for impaired wound healing [58]. The increased ENTPDase activity (Figure 6A) in DW cells therefore indicates a suppressed ATP level and an increased AMP level. The decreased activity of the enzyme in the treated cells indicates a decreased AMP level and an increased ATP level. Increased ATP has been linked to wound closure [59, 60]. Thus, the decreased ENTPDase activity may suggest improved wound healing and corroborates previous reports on improved wound closure with decreased ENTPDase in diabetic mice [58].

The role of Na^+^/K^+^‐ATPase in wound healing has been linked to its mediatory effect on the activation of Src/ERK and PI3K/Akt pathways that promote cell migration and collagen production in fibroblast cells [61]. Thus, the suppressed Na^+^/K^+^‐ATPase activity in DW cells (Figure 6B) correlates with the induction of diabetic wound as evident by the cell gaps (Figure 3). The elevated Na^+^/K^+^‐ATPase activity in DWV1—3 (3, 6 and 12 μg/mL vanillin treatment), DWVL1—3 (PBM + 3, 6 and 12 μg/mL vanillin treatment), and DWL cells may indicate activation of the Src/ERK and PI3K/Akt pathways which suggest wound healing, as evident by the closure of the cell gaps. This correlates with previous studies on the activation of the PI3K/Akt pathway post‐PBM at 660 nm with 5 J/cm^2^ [62].

Increased 5‐LOX activity has been implicated in impaired wound healing in diabetes [63, 64]. This has been attributed to the inflammatory activity of leukotrienes produced from arachidonic acid in a 5‐LOX catalysed reaction [64, 65]. Thus, the elevated 5‐LOX activity in DW cells (Figure 7) indicates an elevated cellular level of leukotrienes, which suggests an occurrence of inflammation. This corroborates previous reports of increased 5‐LOX activity and leukotrienes levels in diabetic wounds [63, 64, 66]. The suppressed 5‐LOX activity in the treated cells, therefore, indicates a reduced cellular level of leukotrienes and an anti‐inflammatory effect. Decreased 5‐LOX activity and leukotrienes levels have been linked with improved wound healing [64, 66], with phenolics reported for their modulatory roles [67].

## 5 Conclusion

Taken together, these results indicate the synergistic therapeutic effect of vanillin and PBM at 660 nm with 5 J/cm^2^ on the management of diabetic wounds, with cotreatment of 6 μg/mL vanillin and PBM displaying the best effect. This is evident by wound closure with improved antioxidative, anti‐inflammatory, purinergic activities, and glucose metabolism.

## Author Contributions

**Ochuko L. Erukainure:** conceptualization (equal), data curation (equal), formal analysis (equal), investigation (equal), methodology (equal), software (equal), writing – original draft (equal). **Nicolette N. Houreld:** conceptualization (equal), funding acquisition (equal), methodology (equal), project administration (equal), resources (equal), software (equal), supervision (equal), validation (equal), writing – review and editing (equal).

## Ethics Statement

The study was approved by the Research Ethics Committee of the Faculty of Health Sciences, University of Johannesburg, Johannesburg, South Africa (Clearance No. REC‐2459‐2023).

## Consent

The authors have nothing to report.

## Conflicts of Interest

The authors declare no conflicts of interest.

## Data Availability

All data used for this study are presented in the article.
